# Evaluation of dietary crude protein concentrations, fishmeal, and sorghum inclusions in broiler chickens offered wheat-based diet via Box-Behnken response surface design

**DOI:** 10.1371/journal.pone.0260285

**Published:** 2021-11-19

**Authors:** Shemil P. Macelline, Peter V. Chrystal, Shiva Greenhalgh, Mehdi Toghyani, Peter H. Selle, Sonia Y. Liu

**Affiliations:** 1 Poultry Research Foundation, The University of Sydney, Camden, NSW, Australia; 2 School of Life and Environmental Sciences, Faculty of Science, The University of Sydney, Sydney, NSW, Australia; 3 Complete Feed Solutions, Hornsby, NSW, Australia; Howick, New Zealand; 4 Sydney School of Veterinary Science, The University of Sydney, Sydney, NSW, Australia; Tokat Gaziosmanpasa Universitesi, TURKEY

## Abstract

The objective of this study was to investigate the impacts of dietary crude protein (CP), fishmeal and sorghum on nutrient utilisation, digestibility coefficients and disappearance rates of starch and protein, amino acid concentrations in systemic plasma and their relevance to growth performance of broiler chickens using the Box-Behnken response surface design. The design consisted of three factors at three levels including dietary CP (190, 210, 230 g/kg), fishmeal (0, 50, 100 g/kg), and sorghum (0, 150, 300 g/kg). A total of 390 male, off-sex Ross 308 chicks were offered experimental diets from 14 to 35 days post-hatch. Growth performance, nutrient utilisation, starch and protein digestibilities and plasma free amino acids were determined. Dietary CP had a negative linear impact on weight gain where the transition from 230 to 190 g/kg CP increased weight gain by 9.43% (1835 versus 2008 g/bird, P = 0.006). Moreover, dietary CP linearly depressed feed intake (r = -0.486. P < 0.001). Fishmeal inclusions had negative linear impacts on weight gain (r = -0.751, P < 0.001) and feed intake (r = -0.495, P < 0.001). There was an interaction between dietary CP and fishmeal for FCR. However, growth performance was not influenced by dietary inclusions of sorghum. Total plasma amino acid concentrations were negatively related to weight gain (r = -0.519, P < 0.0001). The dietary transition from 0 to 100 g/kg fishmeal increased total amino acid concentrations in systemic plasma by 35% (771 versus 1037 μg/mL, P < 0.001). It may be deduced that optimal weight gain (2157 g/bird), optimal feed intake (3330 g/bird) and minimal FCR (1.544) were found in birds offered 190 g/kg CP diets without fishmeal inclusion, irrespective of sorghum inclusions. Both fishmeal and sorghum inclusions did not alter protein and starch digestion rate in broiler chickens; however, moderate reductions in dietary CP could advantage broiler growth performance.

## Introduction

Developing reduced crude protein (CP) diets where soybean meal partially replaced with supplementary amino acids is a promising nutritional strategy to achieve sustainable chicken meat production with reduced nitrogen excretion and improved bird welfare. Dietary CP reduction from 220 to 160 g/kg decreased nitrogen excretion by 35% and dietary CP reduction from 198 to 169 g/kg reduced foot-pad lesion scores by 59% in broiler chickens as reviewed by Greenhalgh et al. [1]. Moreover, a dietary CP reduction from 222 to 165 g/kg reduced soybean meal inclusions by 74% in Chrystal et al. [2]. However, broiler chickens responded to reduced CP diets inconsistently; for instance, broilers offered maize-based, reduced-CP diets improved growth performance in comparison to standard CP diets; whereas, broilers offered wheat-based, reduced-CP diets displayed inferior growth performance [2].

Starch and protein digestive dynamics in broiler chickens are important for optimising growth performance in broiler chickens offered reduced-CP diets because dietary CP reduction increases non-bound amino acid (NBAA) inclusions and starch content [3]. Both glucose and monomeric amino acids are co-absorbed with Na^+^ *via* their respective Na^+^-dependent transport systems; whereas, di- and tri- peptides are absorbed *via* PepT-1 transporter [4, 5]. Glucose and monomeric amino acids may compete for intestinal uptakes [6] and it is possible that such competitions are more pronounced in reduced-CP diets. Liu et al. [7] investigated the influence of diets based on feedstuffs with predetermined starch and protein digestion rate on broiler growth performance from 7 to 35 days post-hatch, where retarding starch digestion rates and/or accelerating protein digestion rates improved FCR and condensed starch:protein digestion rate ratios quadratically (r = 0.648; P < 0.001) improved FCR. In Australia, sorghum is the main alternative feed grain to wheat; starch digestions rates in sorghum are considered slower than wheat under *in vitro* conditions [8]. Moreover, sorghum starch digestion rates were slower than wheat starch by 56% (0.075 versus 0.117 min ^-1^) as determined in broiler chickens in Selle et al. [9]. Fishmeal inclusions at 175 g/kg in sorghum-soybean-meal–based diets significantly increased protein disappearance rates in proximal and distal ileum which indicates that fishmeal can be a more rapidly digestible protein source than soybean meal [10]. Previous studies reported the relevance of protein digestion rate in broiler diet using casein [11] and whey protein concentrate [12]. It is intended to apply a more practical feed ingredient in the present study to test the relevance of starch and protein digestive dynamics. Therefore, the objective of the present study was to evaluate the relevance of starch and protein digestive dynamics in reduced CP diets by varying inclusion levels of fishmeal and sorghum in a wheat-soybean-meal based diets.

Box-Behnken design (BBD) is a multivariate optimization design with the advantage of testing multiple nutrients simultaneously with less number of treatments [13]. It was previously used to optimise digestive dynamics and compare relative importance of dietary factors [14, 15]. Therefore, the experimental diets in the present study contained three levels of dietary CP (190, 210, 230 g/kg), three levels of fishmeal (0, 50, 100 g/kg), and three levels of sorghum (0, 150, 300 g/kg) and response surface was plotted to visualise experimental results. The hypothesis was both starch and protein digestive dynamics, which was reflected by variable dietary fishmeal and sorghum inclusions, would influence growth performance and nutrient utilisation; moreover, it was expected the impact of starch and protein digestive dynamics was more pronounced in diets containing lower CP and higher NBAA.

## Materials and methods

This feeding study was conducted in compliance with the guidelines of the Animal Ethics Committee of The University of Sydney (Project number 2019/1516).

### Experimental design

A three factor, three level Box-Behnken response surface experiment with 13 dietary treatments was used to investigate the impact of three dietary CP levels (190, 210, 230 g/kg) with three inclusion levels of fishmeal (0, 50, 100 g/kg) and sorghum (0, 150, 300 g/kg) on growth performance, nutrient utilisation, starch protein digestibility coefficients and plasma amino acid concentrations from 14 to 35 days post-hatch. The factorial arrangement and their respective levels across 13 dietary treatments are shown in Table 1. The central points were 210 g/kg dietary CP level, 50 g/kg fish meal inclusions, and 150 g/kg sorghum inclusions as described for treatment 13M.

**Table 1:** Factor levels for Box-Behnken design in present study.

Diet	Sorghum (g/kg)	Fishmeal (g/kg)	Dietary crude protein (g/kg)
1A	300	100	210
2B	300	0	210
3C	0	100	210
4D	0	0	210
5E	150	100	230
6F	150	100	190
7G	150	0	230
8H	150	0	190
9I	300	50	230
10J	300	50	190
11K	0	50	230
12L	0	50	190
13M	150	50	210

### Diet preparation

Dietary composition and nutrient specifications are shown in Tables 2 and 3. The nutritionally equivalent diets were formulated based on near-infrared spectroscopy (NIR) of wheat, sorghum and soybean meal using the AMINONir® Advanced program (Evonik Nutrition & Care GmbH, Hanau, Germany). Experimental diets were based on wheat, soybean meal, canola meal with or without sorghum and fishmeal. Varying levels of maize starch ranged from 7.50 to 150 g/kg were added to Diets 1A, 3C, 6F, 8H, 10J, and 12L in order to make all diets iso-energetic (13.0 MJ/kg). Experimental diets were formulated based on digestible amino acids with 10 g/kg of lysine across all dietary treatments. NBAA including lysine, methionine, threonine, tryptophan, valine, arginine, isoleucine, leucine, and glycine were supplemented to maintain similar ideal protein ratios for TSAA, Thr, Trp, Ile, Val, Arg and Gly-equivalent. A commercial starter diet based on wheat and soybean meal with 12.13 MJ/kg energy and 220 g/kg crude protein, was offered to broiler chickens from 1 to 13 days post-hatch. Acid insoluble ash (AIA; Celite^TM^ World Minerals, Lompoc, CA, USA) was included at 20 g/kg in diets as an inert marker in order to determine nutrient digestibility coefficients in two small intestinal sites. Sorghum and wheat were mediumly ground (4.0 mm hammer-mill screen) prior to being blended into the complete diets. All diets were cold-pelleted and contained xylanase and offered to broiler chickens from 14 to 35 days post-hatch.

**Table 2:** Composition of experimental diets.

Ingredients (g/kg)	Experimental diets												
	1A	2B	3C	4D	5E	6F	7G	8H	9I	10J	11K	12L	13M
Wheat	392	301	692	608	492	328	392	525	272	276	578	569	502
Sorghum	300	300	-	-	150	150	150	150	300	300	-	-	150
Maize starch	8.69	-	14.9	-	-	150	-	7.50	-	117	-	128	-
Soybean meal	36.1	172	30.7	163	119	20.1	253	91.5	171	59.1	163	52.4	92.9
Canola meal	83.8	100	81.7	100	56.8	100	72.4	100	100	100	100	100	100
Fishmeal	100	-	100	-	100	100	-	-	50	50	50	50	50
Soybean oil	33.6	62.0	36.0	64.7	42.2	52.9	70.0	51.8	58.8	35.2	61.8	37.1	50.2
*l*-lysine HCl	2.47	3.08	2.48	3.13	0.48	3.30	1.19	5.27	0.73	4.28	0.76	4.32	2.82
*d*,*l*-methionine	1.52	2.05	1.25	1.76	1.00	2.25	1.58	2.43	1.27	2.48	0.97	2.23	1.59
*l*-threonine	1.13	1.06	1.23	1.16	0.33	1.84	0.32	2.06	0.20	1.91	0.29	2.02	1.14
*l*-tryptophan	-	-	0.02	-	-	0.28	-	0.11	-	0.19	-	0.22	-
*l*-valine	0.09	0.14	0.27	0.32	-	1.24	-	1.37	-	1.23	-	1.43	0.19
*l*-arginine	2.66	5.55	2.35	5.16	3.17	3.23	6.11	5.12	4.22	4.36	3.83	4.04	3.75
*l*-isoleucine	0.25	0.11	0.39	0.26	-	1.27	-	1.31	-	1.23	-	1.40	0.27
*l*-leucine	-	-	-	-	-	-	-	-	-	-	-	0.79	-
Glycine	4.33	0.47	3.90	0.04	2.48	5.88	-	2.03	0.74	4.19	0.28	3.80	2.18
Salt	-	0.79	-	0.77	0.73	-	1.51	-	1.14	-	1.12	-	0.37
NaHCO_3_	4.77	4.90	4.68	4.82	3.80	4.78	3.96	5.99	3.73	5.44	3.64	5.34	4.78
Limestone	4.94	1.74	5.04	11.8	4.89	4.78	11.7	12.1	8.01	8.39	8.10	8.47	8.34
MDCP^2^	-	12.4	-	12.3	-	0.36	12.5	13.0	5.44	6.71	5.38	6.68	6.00
Xylanase	0.10	0.10	0.10	0.10	0.10	0.10	0.10	0.10	0.10	0.10	0.10	0.10	0.10
Choline chloride	0.90	0.90	0.90	0.90	0.90	0.90	0.90	0.90	0.90	0.90	0.90	0.90	0.90
Celites	20.0	20.0	20.0	20.0	20.0	20.0	20.0	20.0	20.0	20.0	20.0	20.0	20.0
Sand	-	-	-	-	-	46.8	-	-	-	-	-	-	-
Vit min premix^1^	2.00	2.00	2.00	2.00	2.00	2.00	2.00	2.00	2.00	2.00	2.00	2.00	2.00

^1^Vitamin-trace mineral premix supplied per tonne of feed; [million international units, MIU] retinol 12, cholecalciferol 5, [g] tocopherol 50, menadione3, thiamine 3, riboflavin 9, pyridoxine 5, cobalamin 0.025, niacin 50, pantothenate 18, folate 2, biotin 0.2, copper 20, iron 40 manganese 110, cobalt 0.25, iodine 1, molybdenum 2, zinc 90, selenium 0.3.

^2^MDCP, Mono Dicalcium Phosphate.

**Table 3:** Nutrient specification of experimental diets.

Nutrient (g/kg)	Diet 1A	Diet 2B	Diet 3C	Diet 4D	Diet 5E	Diet 6F	Diet 7G	Diet 8H	Diet 9I	Diet 10J	Diet 11K	Diet 12L	Diet 13M
**Calculated values**													
ME (MJ/kg)	13.0	13.0	13.0	13.0	13.0	13.0	13.0	13.0	13.0	13.0	13.0	13.0	13.0
Crude protein	210	210	210	210	230	190	230	190	230	190	230	190	210
Starch	440	377	440	377	400	428	340	427	360	462	358	463	406
Starch: protein	2.10	1.80	2.10	1.80	1.74	2.25	1.48	2.25	1.57	2.43	1.57	2.44	1.93
Calcium	8.25	8.25	8.25	8.25	8.25	8.25	8.25	8.25	8.25	8.25	8.25	8.25	8.25
Phosphorous available	4.13	4.13	4.13	4.13	4.13	4.13	4.13	4.13	4.13	4.13	4.13	4.13	4.13
Crude fat	67.2	87.2	61.9	82.3	71.4	75.9	89.7	73.7	88.2	64.0	83.6	57.0	76.1
Crude fibre	22.5	25.7	21.3	24.6	20.8	20.4	23.2	24.2	25.8	23.1	24.6	21.5	24.3
*Digestible amino acids*													
Lysine	10.0	10.0	10.0	10.0	10.0	10.0	10.0	10.0	10.0	10.0	10.0	10.0	10.0
TSAA	7.40	7.40	7.40	7.40	7.40	7.40	7.40	7.40	7.40	7.40	7.40	7.40	7.40
Threonine	6.70	6.70	6.70	6.70	6.70	6.70	6.70	6.70	6.70	6.70	6.70	6.70	6.70
Tryptophan	1.90	2.14	1.90	2.11	2.20	1.90	2.42	1.90	2.34	1.90	2.32	1.90	2.01
Isoleucine	7.00	7.00	7.00	7.00	7.75	7.00	7.82	7.00	7.85	7.00	7.72	7.00	7.00
Leucine	13.2	13.5	11.6	11.9	14.1	10.7	14.3	10.8	15.0	11.6	13.5	10.7	12.5
Arginine	10.4	10.4	10.4	10.4	10.4	10.4	10.4	10.4	10.4	10.4	10.4	10.4	10.4
Valine	8.00	8.00	8.00	8.00	8.00	8.84	8.71	8.00	8.95	8.00	8.79	8.00	8.00
Histidine	3.92	4.13	3.97	4.17	4.52	3.41	4.68	3.51	4.63	3.43	4.68	3.46	4.05
Phenylalanine	5.53	8.13	5.35	7.95	6.66	4.25	9.19	6.80	7.96	5.61	7.79	5.39	6.72
Tyrosine	5.43	5.19	5.29	5.03	6.39	4.68	6.11	4.10	6.23	4.46	6.09	4.29	5.19
Proline	9.78	11.8	10.8	12.9	11.1	7.58	13.0	11.6	11.5	9.08	12.5	10.0	11.4
Aspartic acid	3.63	9.26	4.92	10.4	7.88	2.58	1.34	6.81	9.08	4.07	10.3	5.27	6.75
Glutamic acid	17.4	24.0	28.2	34.9	27.3	13.8	33.5	26.5	22.8	14.7	33.7	25.2	25.7
Glycine	8.57	6.83	8.51	6.78	7.74	9.38	7.29	7.62	6.95	8.53	6.89	8.51	7.68
Serine	5.23	7.66	5.30	7.73	6.39	4.08	8.74	6.55	7.49	5.27	7.57	5.31	6.46
Gly- equivalent	12.3	12.3	12.3	12.3	12.3	12.3	12.3	12.3	12.3	12.3	12.3	12.3	12.3
Alanine	1.97	3.98	2.91	4.88	3.73	1.47	5.69	3.38	3.86	1.96	4.78	2.85	3.32
Sodium	1.90	1.90	1.90	1.90	1.90	1.90	1.90	1.90	1.90	1.90	1.90	1.90	1.90
Potassium	4.98	7.33	4.63	6.95	6.32	4.14	8.61	5.59	7.59	5.06	7.22	4.69	5.92
Chloride	1.80	1.80	1.80	1.80	1.80	1.80	1.80	1.80	1.80	1.80	1.80	1.80	1.80
DEB	159	219	150	209	193	135	251	174	226	159	216	150	183
**Analysed values**													
GE (MJ/kg)	16.5	17.1	16.6	17.1	16.7	16.0	17.3	16.8	17.1	16.5	17.2	16.5	17.0
Crude protein	192	201	195	202	219	179	222	183	216	182	223	184	197
Starch	392	351	405	343	369	386	297	360	359	422	322	429	400
Starch: protein	2.04	1.75	2.08	1.70	1.68	2.16	1.34	1.97	1.66	2.32	1.44	2.33	2.03
Total NBAA	12.5	12.5	11.9	11.8	7.47	19.3	9.20	19.7	7.15	19.9	6.13	20.3	11.9

### Bird management

A total of 390 off-sex 14-days old male broilers (Ross 308) were randomly distributed into 65 battery cages each of 6 birds (13 treatments × 5 replicates). The variance of average initial body weight was maintained at 1.02% between cages. The dimensions of the cages were 750 mm in width and depth and 500 mm in height. An environmentally controlled housing facility was used and birds had *ad-libitum* access to feed and water. An initial room temperature of 32 ± 1°C was maintained for the first week, which was gradually decreased to 22 ± 1°C by the end of the third week and maintained at this temperature with a ‘18-hours-on-6-hours-off’ lighting regime for the duration of the feeding study. Initial and final body weights were recorded to determine weight gain. FCR was calculated from feed intake divided by weight gain for the experiment period and any culled/dead bird’s body weights were recorded to adjust feed intakes and FCR calculations.

### Sample collection and chemical analysis

Total excreta were collected from 27 to 29 days post-hatch and feed intake during this period was measured separately to determine apparent metabolizable energy (AME), metabolisable energy to gross energy ratio (ME:GE), N retention and N-corrected apparent metabolisable energy (AMEn). Excreta were dried in a forced-air oven at 80°C for 24 hours and the gross energy **(GE)** of excreta and diets were determined using an adiabatic bomb calorimeter (Parr 1281 bomb calorimeter, Parr Instruments Co., Moline, IL, USA). The AME values (MJ/kg) of the diets were calculated on a dry matter basis from the following equation:

$${AME}_{Diet}=\frac{\left(Feed\;intake\times {GE}_{Diet})-(Excreta\;output\times {GE}_{Excreta}\right)}{\left(Feed\;intake\right)}$$

N contents of diets and excreta were determined using a nitrogen determinator (Leco Corporation, St Joseph, MI) and N retentions calculated from the following equation:

$$Retention\;(\%)=\frac{\left(Feed\;intake\times {Nutrient}_{diet})-(Excreta\;output\times {Nutrient}_{excreta}\right)}{\left(Feed\;intake\times {Nutrient}_{diet}\right)}\times 100$$

N-corrected AME (AMEn MJ/kg DM) values were calculated by correcting N retention to zero using the factor of 36.54 kJ/g N retained in the body [16].

At day 34, three birds at random were selected from each cage and blood samples were taken from the brachial vein to determine the concentrations of 20 amino acids in systemic plasma. Collected blood samples were then centrifuged and the decanted plasma samples were then kept at −80˚C before analysis. Amino acids concentration in systemic plasma was quantified by 6-aminoquinolyl-N-hydroxysuccinimidyl carbamate (AQC; Waters™ AccQTag Ultra; www.waters.com) followed by separation of the derivatives and quantification by reversed phase ultra-performance liquid chromatography [17]. All amino acids were detected by UV absorbance.

On day 35, all birds were euthanized by intravenous injection of sodium pentobarbitone and digesta samples were collected in their entirety from the distal jejunum and distal ileum to determine apparent digestibility coefficients protein (N) and starch in the distal jejunum and ileum. Small intestines were removed from euthanised birds and samples of digesta were gently expressed from the distal jejunum (below the mid-point between duodenal loop and Meckel’s diverticulum) and distal ileum (below the mid-point between Meckel’s diverticulum and the ileo-caecal junction) in their entirety and pooled for each cage. The digesta samples were then freeze-dried. Starch concentrations in diets and digesta were determined by a procedure based on dimethyl sulphoxide, α-amylase and amyloglucosidase as described by Mahasukhonthachat et al. [18]. N concentrations were determined as already stated and AIA concentrations were determined by the method of Siriwan et al. [19]. The apparent digestibility coefficients for starch and protein (N) in two small intestinal sites were calculated from the following equation:

$$Digestibility\;Coefficient=\frac{{\left(Nutrient/AIA\right)}_{diet}-{(Nutrient/AIA)}_{digesta}}{{\left(Nutrient/AIA\right)}_{diet}}$$

Starch and protein (N) disappearance rates (g/bird/day) were deduced from the following equation:

Dissaperencerate=Feedintake×Dietarynutrientconcentration×digestibilitycoefficient

### Statistical analyses

The experimental units were replicate cage means (6 birds per cage) and statistical procedures included model prediction and linear regression analysis. The surface plots of 3-factor, 3-level Box-Behnken design were obtained by R 3.5.3 software. Best fitted models for response surface designs were predicted by combinations of first- and second-degree polynomial regressions. In model prediction, non-significant coefficients were omitted and the equations were re-predicted with only significant coefficients for each response variable. When more than one models were fitted and significantly different, “Akaike Information Criterion” was used for model comparison and selection. Solver function in Microsoft excel was used to calculate best dietary CP levels with optimal inclusions of fishmeal and sorghum based on response surface design equations.

## Results

The influence of dietary treatments on weight gain, feed intake, FCR, and relative fat pad weights are shown in Table 4. The overall average weight gain and feed intake for all treatments from 14 to 35 days post-hatch were higher than 2019 Ross performance objectives by 3.41% (1912 versus 1849 g/bird) and 4.48% (3052 versus 2921 g/bird), respectively and FCR was comparable (1.601 versus 1.579). The mortality rate in the present study was 1.28% which was not influenced by the dietary treatments (P = 0.77).

**Table 4:** Effect of dietary treatments on growth performance, and relative abdominal fat-pad weights in broiler chickens from 14 to 35 days post-hatch.

Diet	Weight gain (g/bird)	Feed intake (g/bird)	FCR (g/g)	Relative fat-pad weights (g/kg)
1A	1764	2883	1.642	13.3
2B	2053	3160	1.540	12.7
3C	1682	2783	1.655	12.7
4D	2063	3144	1.525	9.74
5E	1678	2889	1.723	12.7
6F	1875	3158	1.685	16.0
7G	2002	3128	1.563	10.3
8H	2140	3270	1.529	13.0
9I	1800	2934	1.630	11.4
10J	1953	3129	1.602	15.8
11K	1857	2901	1.561	11.0
12L	2064	3267	1.583	14.6
13M	1925	3024	1.571	12.9
**Crude protein (g/kg)**				
190	2008	3206	1.600	14.9
210	1897	2999	1.587	12.3
230	1835	2963	1.619	11.3
Linear relationship (r =)	-0.414	-0.486	0.094	-0.620
P-value	0.006	<0.0001	0.457	<0.0001
**Sorghum (g/kg)**				
0	1917	3024	1.581	12.0
150	1924	3094	1.614	13.0
300	1893	3027	1.603	13.3
Linear relationship (r =)	-0.058	0.005	0.108	0.228
P-value	0.648	0.967	0.393	0.068
**Fishmeal (g/kg)**				
0	2065	3176	1.539	11.4
50	1920	3051	1.589	13.2
100	1750	2928	1.676	13.7
Linear relationship (r =)	-0.751	-0.495	0.660	0.395
P-value	<0.0001	<0.0001	<0.001	0.0011

**Fig 1:**
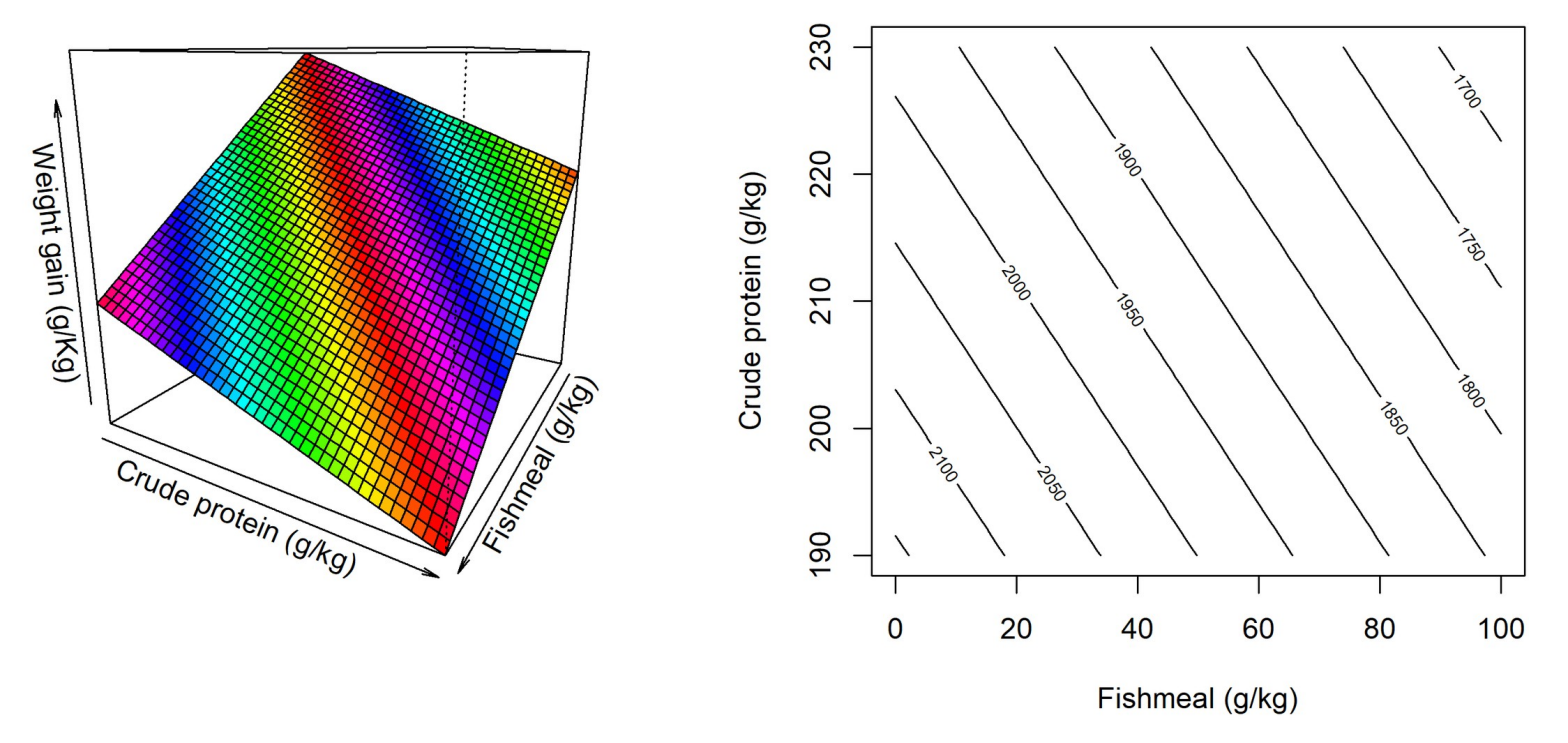
Response surface and contour plot describing influence of fishmeal inclusion and dietary crude protein level on weight gain in broiler chickens from 14–35 days post-hatch.

The response surface and contour plot for weight gain is illustrated in Fig 1 and there was no interaction between dietary factors. However, increasing dietary CP and fishmeal inclusions linearly reduced the weight gain regardless of the sorghum inclusion. According to the coefficient of the equation, the negative impact of dietary CP was greater than fishmeal inclusion, as described by the following equation,

$$Weight\;Gain\;(g)=2982-4.3425\;CP-3.1510\;FM,\;(R^{2}=0.736,\;P<0.001).$$

Based on the above equation, the optimal weight gain of 2157 g/bird was predicted with 190 g/kg dietary CP and no fishmeal regardless of sorghum inclusions.

**Fig 2:**
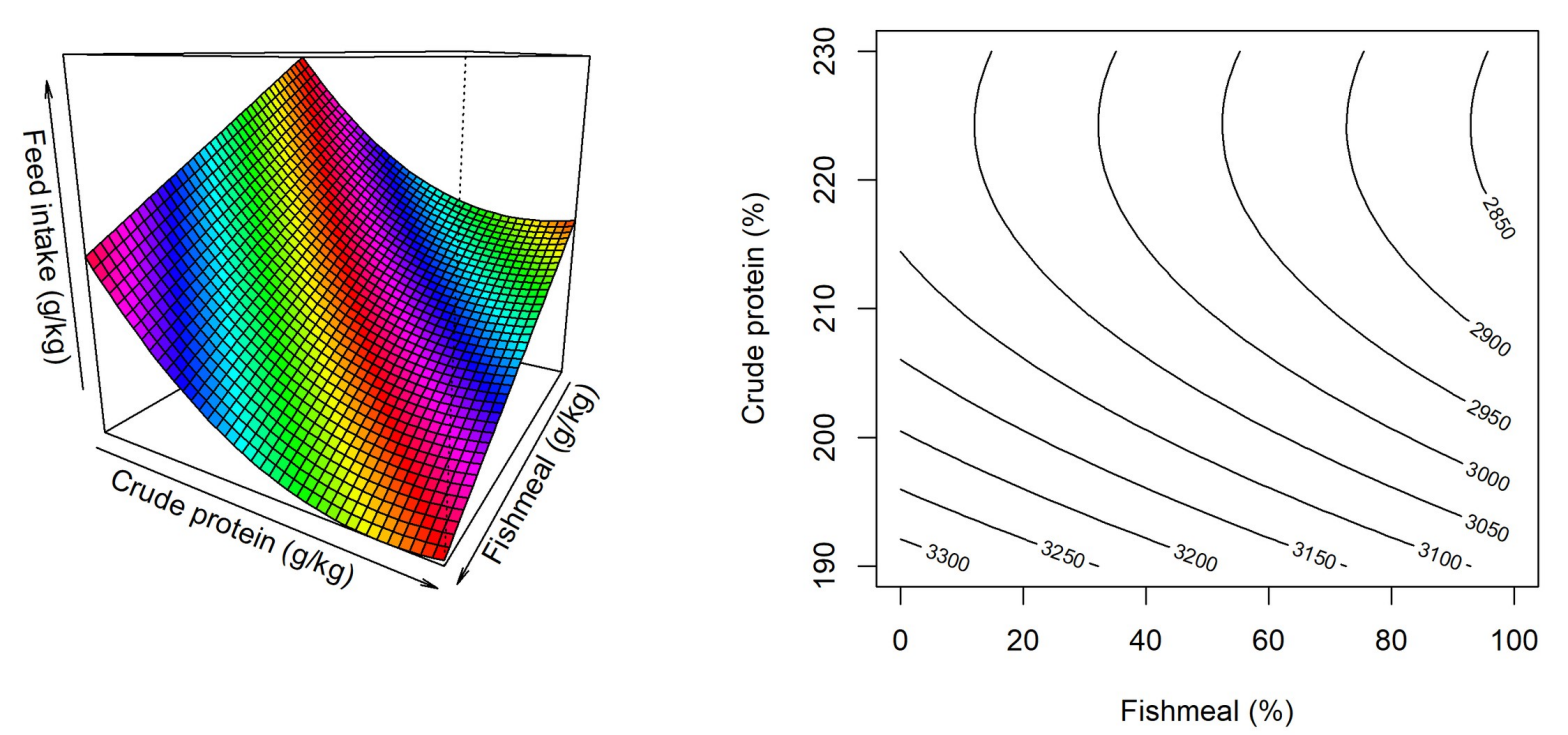
Response surface and contour plot describing influence of fishmeal inclusion and dietary crude protein level on feed intake in broiler chickens from 14–35 days post-hatch.

The response surface for feed intake is illustrated in Fig 2. Only fishmeal inclusion and dietary CP level influenced feed intake where increasing fishmeal inclusion depressed feed intake and dietary CP had quadratic relationship with feed intake. The predicted equation for the feed intake is,

$$Feed\;intake\;(g)=13806-2.472\times FM-95.68\times CP+2.133\times {CP}^{2},\;(R^{2}=0.503,\;P<0.001).$$

**Fig 3:**
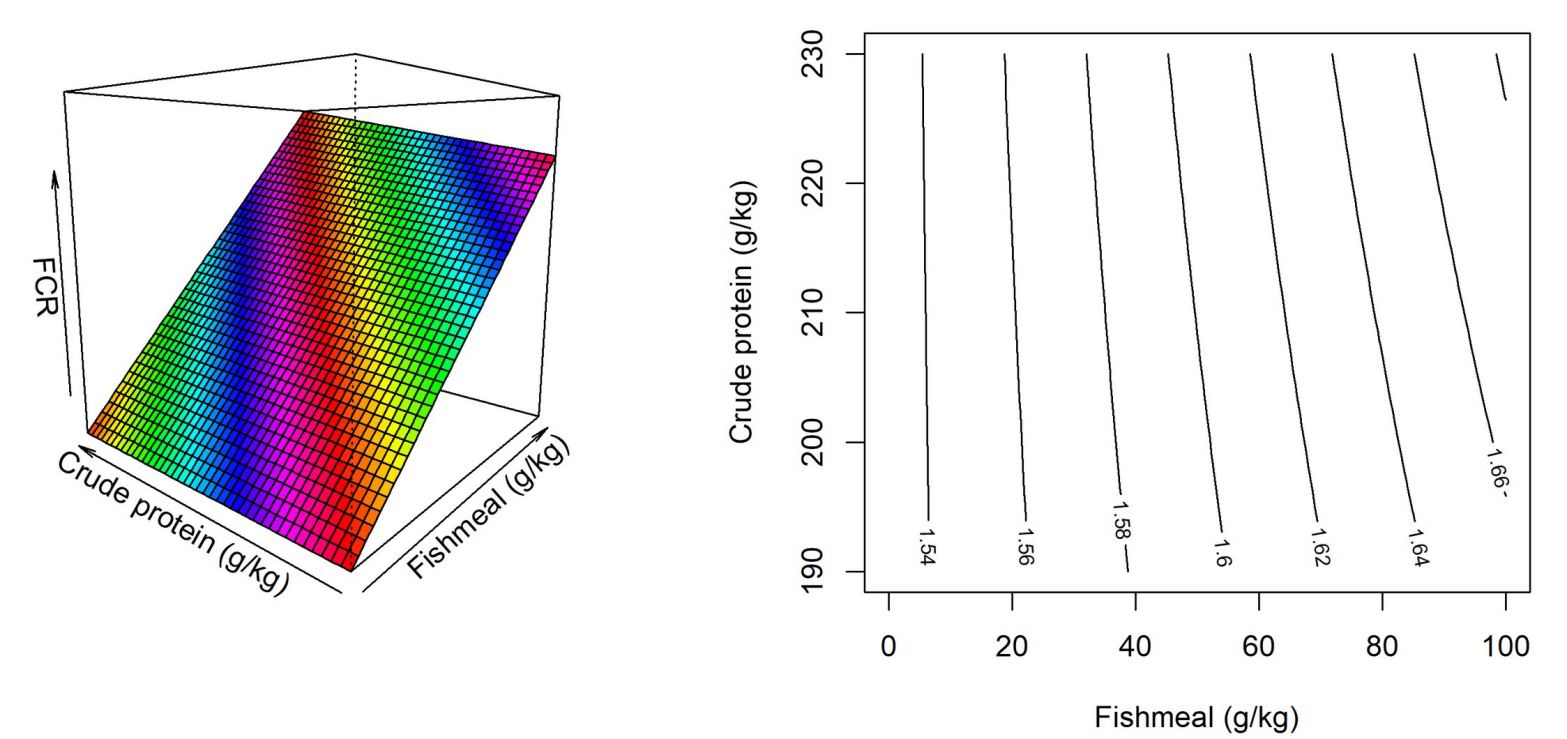
Response surface and contour plot describing influence of fishmeal inclusion and dietary crude protein level on FCR in broiler chickens from 14–35 days post-hatch.

The response surface and contour plot for FCR is illustrated in Fig 3. There was a CP and fishmeal interaction on FCR. That negative impact of fish meal inclusions on FCR was more pronounced in diets containing 230 g/kg CP than diets containing 190 g/kg CP. The response of FCR was described by the following equation,

$$FCR=1.532+6.54\times 10^{-6}\times CP\times FM,\;(R^{2}=0.434,\;P<0.001).$$

**Fig 4:**
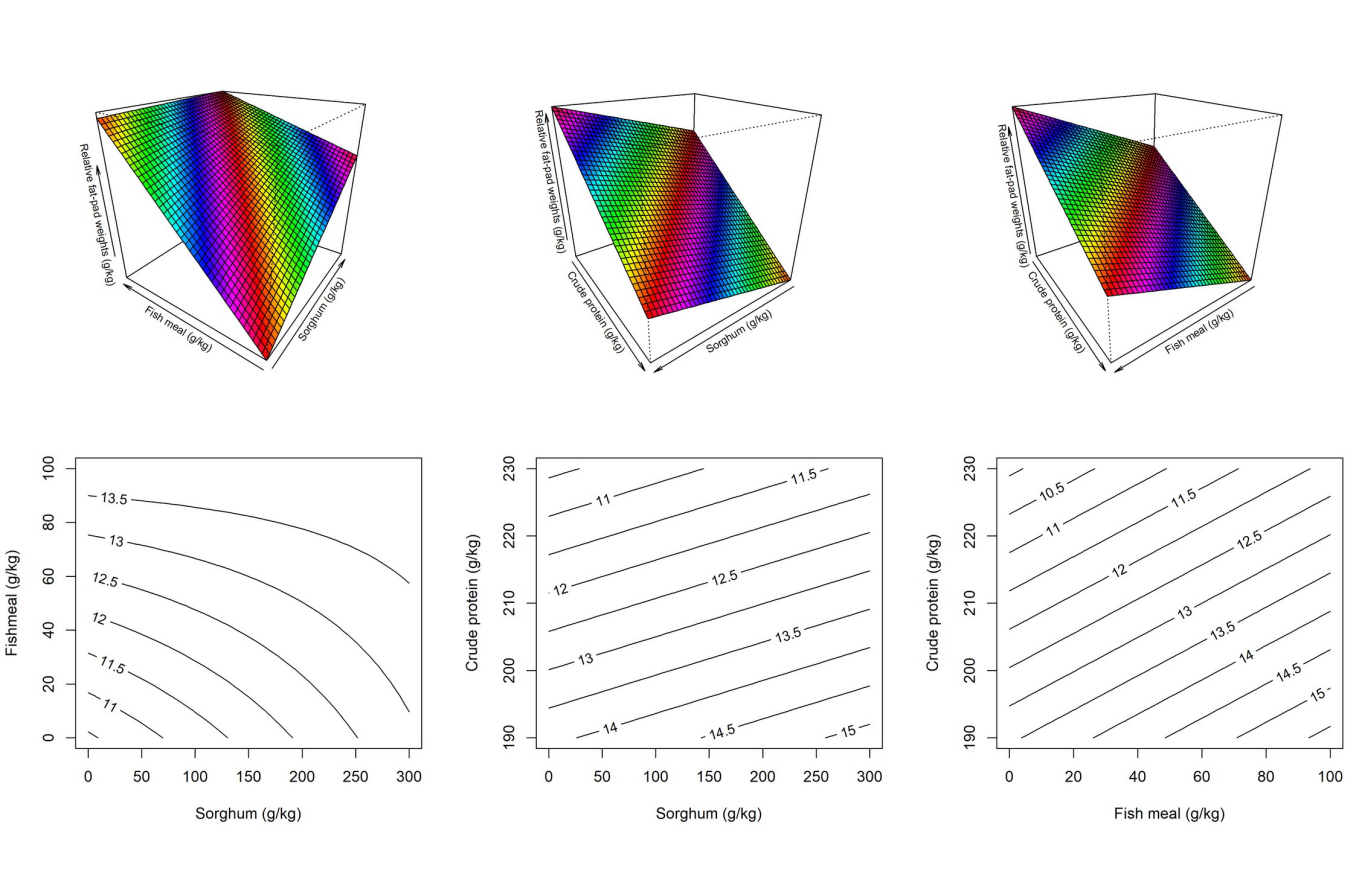
The response surface designs and contour plots describing the impact of dietary crude protein level together with sorghum and fishmeal inclusions on relative fat pad weights in broiler chickens at 35 days post-hatch (left–slice at 210 g/kg of CP; middle–slice at 5 g/kg of FM; right–slice at 150 g/kg of sorghum).

Fig 4 illustrates response surface and contour plots describing the relationship between fish meal inclusions, sorghum inclusions and dietary CP with relative fat pad weights. The surface design shows that increasing fish meal and sorghum inclusions increased the relative fat pad weights whereas dietary CP had negative impact. The predicted equation for relative fat pad weight is as follows,

$$Relative\;fat\;pad\;weight\;(g)=28.8552+0.03418\times FM-0.08776\times CP+0.00824\times Sorghum-7.9\times 10^{-5}\;FM\times Sorghum,\;(R^{2}=0.614,\;P<0.001).$$

The predicted lowest relative fat pad weight of 8.67 g/kg was obtained at 230 g/kg dietary CP with no inclusion of fish meal and sorghum.

**Fig 5:**
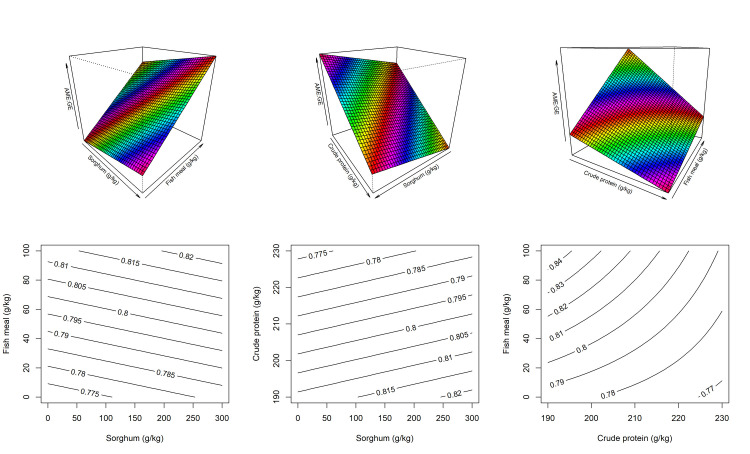
The response surface designs and contour plots describing the impact of dietary crude protein level with sorghum and fishmeal inclusions on AME:GE ratio in broiler chickens in 27–29 days post-hatch (left–slice at 210 g/kg of CP; middle–slice at 5 g/kg of FM; right–slice at 150 g/kg of sorghum).

The impact of dietary treatments on nutrient utilization including AME, ME:GE, nitrogen retention and N corrected apparent metabolisable energy in broiler chickens from 27–29 post-hatch is reported in Table 5. The response surface and contour plots for ME:GE ratio is illustrated in Fig 5. Increasing fishmeal and sorghum inclusion and decreasing dietary CP resulted higher ME:GE ratio. There is an interaction between fishmeal and CP on ME:GE ratio where higher inclusion of fishmeal to reduced CP diets increased ME:GE to larger extent than under high CP diets (Fig 5 right). The predicted equation for response surface design for ME:GE ratio as follows,

$$ME:GE=0.86+2.625\times 10^{-3}\times FM+3.50\times 10^{-5}\times Sorghum-4.38\times 10^{-4}\times CP-1.05\times 10^{-5}\times FM\times CP,\;(R^{2\;}=0.711,\;P<0.0001)$$

**Table 5:** Effect of dietary treatments on nutrient utilisation (AME: Apparent metabolisable energy, ME:GE: Metabolisable to gross energy ratio, N: Nitrogen retention, AMEn: N-corrected AME) in broiler chicken from 27 to 29 days post-hatch.

Diet	AME (MJ/kg)	ME:GE ratio	N retention (%)	AMEn (MJ/kg)
1A	15.49	0.82	65.52	14.38
2B	15.46	0.79	66.81	14.14
3C	15.53	0.82	65.93	14.45
4D	15.04	0.77	61.78	13.81
5E	15.26	0.80	63.73	14.04
6F	15.45	0.85	71.58	14.22
7G	15.32	0.77	62.21	13.99
8H	15.13	0.78	7076	13.86
9I	15.23	0.77	60.93	14.05
10J	15.60	0.83	69.62	14.44
11K	14.99	0.77	61.09	13.79
12L	15.20	0.81	68.78	13.92
13M	15.35	0.80	64.10	14.21
**Crude protein (g/kg)**				
190	15.34	0.82	70.18	13.97
210	15.37	0.80	64.83	14.20
230	15.20	0.78	61.99	13.96
Linear relationship (r =)	-0.185	-0.554	-0.709	-0.160
P-value	0.140	<0.0001	<0.0001	0.203
**Sorghum (g/kg)**				
0	15.19	0.79	64.40	13.99
150	15.30	0.80	66.48	14.06
300	15.44	0.80	65.72	14.26
Linear relationship (r =)	0.319	0.126	0.115	0.303
P-value	0.009	0.310	0.364	0.014
**Fishmeal (g/kg)**				
0	15.23	0.78	65.39	13.95
50	15.27	0.82	64.90	14.08
100	15.43	0.80	66.69	14.27
Linear relationship (r =)	0.247	0.598	0.113	0.368
P-value	0.047	<0.001	0.372	0.003

The highest predicted ME:GE ratio of 0.85 was estimated at 190 g/kg of dietary CP level without fish meal and sorghum inclusions.

The effect of dietary treatments on apparent digestibility coefficients and disappearance rates of starch in distal jejunum and distal ileum at 35 days post-hatch is reported in Table 6. Average starch digestibility coefficients in distal jejunum and distal ileum are 0.966 and 0.995 respectively. Crude protein concentrations linearly decreased starch disappearance rate in the distal jejunum (r = -0.800, P < 0.001) and distal ileum (r = -0.812, P < 0.001). Sorghum inclusions had no impact on starch digestibility and disappearance rate. Fishmeal inclusions slightly increased starch digestibility in distal ileum from 0.995 to 0.998 (r = 0.334, P = 0.009). The results of Apparent digestibility coefficients, disappearance rates of protein and starch: protein disappearance rate ratios in distal jejunum and distal ileum are shown in Table 7. Dietary crude protein did not influence apparent protein digestibility coefficients in the distal jejunum and ileum; but it linearly reduced starch and protein disappearance rate ratios in the distal jejunum (r = -0.639, P < 0.001) and ileum (r = -0.779, P < 0.001). Sorghum inclusions had no impact on apparent jejunal protein digestibilities but increasing sorghum inclusion linearly decreased ileal protein digestibility (r = -0.260, P = 0.036). Fishmeal inclusion also had no impact on apparent protein digestibility coefficients in the distal jejunum and ileum. However, increasing fishmeal inclusion increased starch and protein disappearance rate ratios in the distal jejunum (r = 0.342, P = 0.005) and distal ileum (r = 0.375, P = 0.005).

**Table 6:** Effect of dietary treatments on apparent starch digestibility coefficients and disappearance rates in distal jejunum and distal ileum at 35 days post-hatch.

Diet	Digestibility coefficients		Disappearance rates (g/bird/day)	
	Distal jejunum	Distal ileum	Distal jejunum	Distal ileum
1A	0.969	0.999	60.0	62.2
2B	0.970	0.997	58.4	60.5
3C	0.966	0.999	59.4	61.4
4D	0.968	0.996	56.8	59.5
5E	0.964	0.996	56.2	58.1
6F	0.968	0.998	64.2	67.4
7G	0.968	0.970	48.9	50.0
8H	0.951	0.997	61.1	64.0
9I	0.970	0.997	56.0	57.5
10J	0.971	0.998	69.9	71.9
11K	0.964	0.997	48.6	49.2
12L	0.965	0.999	73.6	76.1
13M	0.964	0.997	63.0	65.2
**Crude protein (g/kg)**				
190	0.964	0.998	67.2	70.0
210	0.967	0.998	59.6	61.8
230	0.967	0.995	52.4	59.9
Linear relationship (r =)	0.094	-0.284	-0.800	-0.812
P-value	0.455	0.027	<0.0001	<0.0001
**Sorghum (g/kg)**				
0	0.966	0.998	59.6	62.4
150	0.963	0.995	58.7	60.1
300	0.970	0.998	61.2	63.1
Linear relationship (r =)	0.129	0.004	0.086	0.071
P-value	0.306	0.978	0.496	0.589
**Fishmeal (g/kg)**				
0	0.964	0.995	56.4	58.4
50	0.967	0.998	62.2	64.6
100	0.967	0.998	59.9	62.0
Linear relationship (r =)	0.071	0.334	0.190	0.190
P-value	0.577	0.009	0.130	0.143

**Table 7:** Effect of dietary treatments on apparent crude protein digestibility coefficients, disappearance rates and starch: Protein disappearance rate ratios in distal jejunum and distal ileum at 35 days post-hatch.

Diet	Digestibility coefficients		Disappearance rates (g/bird/day)		Starch: protein disappearance rate ratio	
	Distal jejunum	Distal ileum	Distal jejunum	Distal ileum	Distal jejunum	Distal ileum
1A	0.618	0.725	18.69	22.01	3.27	2.80
2B	0.598	0.723	20.84	25.14	2.93	2.41
3C	0.623	0.736	18.39	21.76	3.27	2.82
4D	0.610	0.706	21.15	24.53	2.73	2.30
5E	0.586	0.738	20.23	25.51	2.79	2.28
6F	0.528	0.727	16.31	22.30	4.15	2.96
7G	0.578	0.671	21.87	25.37	2.31	1.99
8H	0.619	0.749	20.21	24.50	3.05	2.62
9I	0.607	0.697	21.05	24.20	2.69	2.39
10J	0.583	0.703	18.14	21.83	3.90	3.30
11K	0.649	0.774	22.60	26.96	2.16	1.89.
12L	0.660	0.766	21.57	25.02	3.43	3.05
13M	0.618	0.752	19.93	24.12	3.20	2.70
**Crude protein (g/kg)**						
190	0.598	0.736	19.06	23.41	3.63	2.98
210	0.613	0.729	19.80	23.53	3.08	2.61
230	0.604	0.720	21.44	25.51	2.49	2.15
Linear relationship (r =)	0.033	-0.128	0.292	0.354	-0.639	-0.779
P-value	0.796	0.321	0.019	0.004	<0.0001	<0.0001
**Sorghum (g/kg)**						
0	0.636	0.746	20.93	24.57	2.90	2.56
150	0.586	0.728	19.71	24.38	3.10	2.49
300	0.602	0.712	19.68	23.29	3.20	2.72
Linear relationship (r =)	-0.160	-0.260	-0.153	-0.214	0.167	0.169
P-value	0.203	0.036	0.223	0.087	0.184	0.226
**Fishmeal (g/kg)**						
0	0.601	0.713	21.01	24.89	2.76	2.33
50	0.623	0.738	20.66	24.44	3.08	2.70
100	0.589	0.732	18.41	22.89	3.37	2.70
Linear relationship (r =)	-0.058	0.147	-0.321	-0.336	0.342	0.375
P-value	0.643	0.241	0.009	0.006	0.005	0.005

The impact of dietary treatments on free amino acids concentrations in the systemic plasma is shown in Tables 8 and 9. Dietary crude protein linearly increased plasma concentrations of Arg, His, Ile, Leu, Phe, Asn+Asp and Tyr. Dietary crude protein linearly decreased plasma concentrations of Met, Thr, Glu+Gln and Gly. Sorghum inclusions linearly decreased plasma concentrations of Leu, Val and Tyr. Fishmeal inclusions linearly increased plasma concentrations of His, Ile, Leu, Met, Phe, Thr, Val, Ala, Cys, Glu+Gln, Gly, Pro and Ser; but decreased plasma concentrations of Arg, Lys and Asn + Asp.

**Table 8:** Effects of dietary treatments on free essential amino acid systemic plasma concentrations (μg/mL) at 34 days post-hatch.

Diet	Arg	His	Ile	Leu	Lys	Met	Phe	Thr	Trp	Val
1A	77.5	20.5	11.4	29.0	14.5	15.5	22.4	109.3	4.6	23.0
2B	119.2	8.1	9.8	22.5	17.8	10.8	19.0	58.6	4.9	18.4
3C	66.9	21.8	14.2	23.7	13.8	14.5	22.7	106.1	5.0	28.9
4D	110.6	8.7	11.2	18.2	22.0	10.0	17.0	64.3	4.8	21.2
5E	106.5	26.2	16.1	31.7	15.5	12.6	24.0	92.3	5.4	31.9
6F	58.8	5.5	13.9	20.3	15.2	17.6	17.0	114.2	5.4	30.8
7G	106.5	13.8	12.4	23.1	17.2	8.3	20.0	54.5	5.3	22.7
8H	100.6	5.0	12.1	15.8	21.9	14.2	16.2	72.5	5.1	24.6
9I	116.2	21.4	14.8	31.9	16.7	12.0	23.5	73.2	5.6	29.1
10J	87.8	9.6	12.1	22.3	17.4	17.3	19.8	100.6	4.9	25.8
11K	120.5	22.1	15.5	26.2	17.5	10.8	22.1	74.3	5.5	30.2
12L	76.9	5.2	13.9	17.9	15.6	15.1	18.0	102.7	5.3	31.1
13M	100.6	13.9	11.1	23.1	14.2	12.0	20.4	80.5	5.00	21.9
**Linear relationships**										
**Crude protein (g/kg)**										
190	81.0	6.3	13.0	19.1	17.5	16.1	17.8	97.5	5.2	28.1
210	95.0	14.6	11.5	23.3	16.5	12.5	20.3^b^	83.8	4.9	22.6
230	112.4	20.9	14.7	28.2	16.7	10.9	22.4	73.6	5.4	28.5
r =	0.453	0.726	0.267	0.661	-0.066	-0.598	0.614	-0.424	0.177	0.029
P =	<0.0001	<0.001	0.032	<0.001	0.602	<0.0001	<0.001	<0.001	0.160	0.821
**Sorghum (g/kg)**										
0	93.7	14.5	13.7	21.5	17.2	12.6	20.0	86.8	5.1	27.8
150	94.6	12.9	13.1	22.8	16.8	13.0	19.5	82.8	5.2	26.4
300	100.2	14.9	12.0	26.4	16.6	13.9	21.2	85.4	5.0	24.1
r =	0.093	0.022	-0.258	0.357	-0.047	0.154	0.159	-0.025	-0.081	-0.275
P =	0.460	0.864	0.038	0.004	0.711	0.221	0.206	0.842	0.521	0.026
**Fishmeal (g/kg)**										
0	109.2	8.9	11.4	19.9	19.7	10.8	18.1	62.5	5.0	21.7
50	100.4	14.5	13.5	24.3	16.3	13.4	20.7	86.3	5.3	27.6
100	77.4	18.5	13.9	26.2	14.7	15.1	21.5	105.5	5.1	28.6
r =	-0.459	0.477	0.393	0.450	-0.397	0.492	0.460	0.761	0.055	0.504
P =	<0.001	<0.001	0.001	0.0002	0.001	<0.001	0.001	<0.001	0.665	<0.001

**Table 9:** Effects of dietary treatments on free non-essential and total amino acid systemic plasma concentrations (μg/mL) at 34 days post-hatch.

Diet	Ala	Asn+Asp	Cys	Glu+Gln	Gly	Pro	Ser	Tyr	Total
1A	78.9	94.7	16.0	242.2	138.9	72.4	83.2	40.7	1036.0
2B	65.2	137.1	15.9	190.8	47.7	41.7	54.7	41.7	775.2
3C	72.8	79.9	18.5	277.0	127.3	81.8	76.5	37.1	1042.2
4D	59.8	130.4	15.1	196.4	43.8	45.6	54.4	34.2	763.4
5E	77.7	122.4	17.4	223.1	105.4	78.7	80.4	43.0	1025.2
6F	78.7	73.9	16.7	274.8	165.4	57.7	89.6	29.7	1044.9
7G	58.7	123.1	14.8	181.0	46.9	45.0	54.3	38.3	751.8
8H	68.5	114.5	14.7	221.4	48.9	48.3	49.6	28.6	793.0
9I	77.8	134.7	15.8	202.8	76.0	60.1	58.1	47.3	919.3
10J	74.5	105.5	15.6	243.7	110.2	57.8	72.5	30.9	954.8
11K	71.2	137.4	16.9	202.3	71.4	66.3	64.8	39.7	912.2
12L	72.6	97.3	17.0	283.5	100.5	59.4	72.3	26.8	963.8
13M	68.3	116.5	15.7	208.2	81.3	59.2	61.1	37.5	864.6
**Linear relationships**									
**Crude protein (g/kg)**									
190	73.6	97.8	16.0	255.9	106.3	55.8	71.0	29.0	939.1
210	69.0	111.7	16.4	222.9	87.8	60.1	66.0	38.4	896.4
230	71.4	129.4	16.2	202.3	74.9	62.5	64.4	42.1	902.1
r =	-0.076	0.448	0.054	-0.434	-0.301	0.175	-0.187	0.736	-0.105
P =	0.135	0.0002	0.671	0.0003	0.015	0.164	0.135	<0.001	0.405
**Sorghum (g/kg)**									
0	69.1	111.3	16.8	239.8	85.7	62.3	67.0	34.4	920.5
150	70.4	110.1	15.9	221.7	89.6	57.8	67.0	35.4	895.9
300	74.1	118.0	16.0	219.9	93.2	58.0	67.1	40.2	921.3
r =	0.171	0.095	-0.197	-0.162	-0.197	-0.137	0.003	0.323	0.002
P =	0.173	0.450	0.116	0.198	0.572	0.276	0.982	0.009	0.985
**Fishmeal (g/kg)**									
0	63.0	126.3	15.1	197.4	46.8	45.1	53.3	35.7	770.8
50	72.9	118.3	16.2	228.1	87.9	60.6	65.8	36.5	922.9
100	77.0	92.7	17.4	254.3	134.3	72.7	82.4	37.6	1037.2
r =	0.479	-0.478	0.554	0.461	0.839	0.715	0.830	0.110	0.756
P =	<0.001	<0.0001	<0.001	0.0001	<0.001	<0.000	<0.001	0.384	<0.001

## Discussion

In the present study, dietary CP reductions from 230 to 210 and 190 g/kg linearly increased weight gain (r = 0.414, P < 0.01) and feed intake (r = 0.486, P < 0.001) without influencing FCR. Accordingly, birds offered 190 g/kg CP diets had 9.43% (2008 versus 1835 g/bird, P < 0.05) higher weight gain and 8.20% (3206 versus 2963 g/bird, P < 0.05) higher feed intake compared to birds offered 230 g/kg CP diets. However, the 190 g/kg CP diet represents a modest reduction in dietary CP for birds from 14 to 35 days post-hatch. Chrystal et al. [20] reported modest reduction in dietary CP increased weight gain by 8.22% (2396 versus 2214 g/bird) and reduced FCR 2.62% (1.453 versus 1.415) from 7–35 days post-hatch. Thus, birds have the potential to perform satisfactorily when offered moderately reduced-CP diets which was consistent with the present study. However, more tangible reductions in dietary CP usually compromise growth performance with an increase in carcass fat deposition [21, 22]. In the present study, on average, transition of dietary CP from 230 to 190 g/kg reduced soybean meal inclusions (56 versus 177 g/kg) whilst increasing NBAA inclusions (7.49 versus 19.8 g/kg). This outcome supports the rationale in Selle et al. [23] that replacing soybean meal with NBAA in reduced-CP diets could be a promising strategy to reduce the chicken-meat industry’s dependence on soybean meal. However, further reductions of dietary CP requires higher inclusions of NBAA as evident in Chrystal et al. [2]. This may generate amino acid imbalances at sites of protein synthesis; if so, increased deamination would generate ammonia and may compromise broiler growth performance [24].

Fishmeal is usually recognised as a prolific source of digestible amino acids in broiler chickens [25] and pigs [26] and was included in the present experiment to diversify the rate of protein digestion in order to investigate the influence of protein and starch digestive dynamics in diets containing different CP levels. However, it was not anticipated that the 100 g/kg fish meal inclusion would significantly compromise growth performance in the present study. This is in complete contrast to the positive growth performance responses generated by 175 g/kg fishmeal inclusions in sorghum-based broiler diets observed by Sydenham et al. [10]. Nevertheless, the unexpected impact of fishmeal is not without precedent. It was reported decades ago [27] that overheating fishmeal during the rendering process depressed the availability of amino acids in broiler chickens. Lysine and aspartic acid, in particular, appear to be vulnerable to improper processing in both fishmeal and other protein meals [28–30]. In the present study, fishmeal inclusion negatively influenced plasma concentrations of Lys (r = -0.397, P = 0.001) and Asn + Asp (r = -0.478, P < 0.001). Unfortunately, due to the lack of variable impact of sorghum and fishmeal inclusions on distal jejunal starch and protein digestibilities, respectively, the relevant importance of starch and protein digestive dynamics in diets containing different CP was not tested as originally planned. However, attention needs to be drawn on inconsistent growth performance in broiler chickens when offered diets containing sorghum and fishmeal.

Sorghum has been associated with sub-optimal growth performance in broiler chickens, consequently, its inclusion in practical diets is often limited. However, in the present study, the substitution of wheat with sorghum did not influence broiler performance, which implies the two feed grains used in the present study were nutritionally equivalent where broilers offered diets with 0 and 300 g/kg sorghum generated comparable starch digestibility coefficients in distal jejunum (0.966 versus 0.970). This suggests that the starch digestibility/energy utilization of sorghum used in the present study was of an unusually high order. Wheat is commonly recognised as a better feed grain under Australian conditions, but this is not necessarily always the case. The performance of broilers offered maize-, sorghum- and wheat-based diets, without and with exogenous phytase, was compared from 1 to 27 days post-hatch in Liu et al. [31]. As main effects, sorghum supported significantly better weight gain by 6.70% (1338 versus 1254 g/bird) and FCR by 3.60% (1.471 versus 1.526) than wheat-based diets. Alternatively, Moss et al. [32] compared two sorghum-based diets with two wheat-based diets offered to broilers from 1 to 35 days post-hatch. While average weight gains were nearly identical (2670 versus 2676 g/bird), birds offered wheat-based diets enjoyed an advantage of 3.41% (1.415 versus 1.465) in FCR. Thus, the relative nutritional values of the two feed grains are sufficiently variable that the outcome from any one comparison cannot be predicted with any accuracy. The comparable quality of sorghum starch to wheat starch in the present study failed to generate different starch digestion rates.

The Box-Behnken design is challenged in the present study because there are additional variations in dietary compositions to the three factors being evaluated. Concentrations of soybean meal ranged from 20.1 to 253 g/kg and NBAA from 6.13 to 19.90 g/kg across the 13 dietary treatments (Table 2). Interestingly, significant multiple linear regressions were detected for feed intakes (r = 0.746; P < 0.0001) and weight gains (r = 0.860; P < 0.0001) when soybean meal and NBAA were considered in addition to fishmeal. The equations are as follows:

$$Feed\;intake_{\left(g/bird\right)}=2402–0.247\times FM_{\left(g/kg\right)}+1.718\times SBM_{\left(g/kg\right)}+36.36\times NBAA_{\left(g/kg\right)}$$

$$Weight\;gain_{\left(g/bird\right)}=1849–3.016\times FM_{\left(g/kg\right)}+0.05\times SBM_{\left(g/kg\right)}+14.5\times NBAA_{\left(g/kg\right)}.$$

Thus, in both instances, inclusions of soybean meal and NBAA promoted feed intakes and weight gains; whereas, fishmeal inclusions had negative impacts on growth performance.

## Conclusions

Growth performance were compromised by higher fishmeal inclusion and was not influenced by sorghum substitution. Both fishmeal and sorghum inclusions did not alter protein and starch digestion rate in broiler chickens; however, it is evident that moderate reductions in dietary CP could advantage broiler growth performance.

## Supporting information

S1 Raw data (XLSX)
